# The Involvement of Ethylene in Calcium-Induced Adventitious Root Formation in Cucumber under Salt Stress

**DOI:** 10.3390/ijms20051047

**Published:** 2019-02-28

**Authors:** Jian Yu, Lijuan Niu, Jihua Yu, Weibiao Liao, Jianming Xie, Jian Lv, Zhi Feng, Linli Hu, Mohammed Mujitaba Dawuda

**Affiliations:** 1 College of Horticulture, Gansu Agricultural University, Lanzhou 730070, China; Yjian0515@163.com (J.Y.); niulj0508@163.com (L.N.); liaowb@gsau.edu.cn (W.L.); xiejianming@gsau.edu.cn (J.X.); lvjian@gsau.edu.cn (J.L.); fengz@gsau.edu.cn (Z.F.); hull@gsau.edu.cn (L.H.); 2 Horticulture Department, FoA University For Development Studies, Box TL 1350 Tamale, Ghana; mmdawuda@yahoo.com

**Keywords:** calcium, ethylene, adventitious rooting, ethylene biosynthesis, salt stress

## Abstract

Calcium and ethylene are essential in plant growth and development. In this study, we investigated the effects of calcium and ethylene on adventitious root formation in cucumber explants under salt stress. The results revealed that 10 μM calcium chloride (CaCl_2_) or 0.1 μM ethrel (ethylene donor) treatment have a maximum biological effect on promoting the adventitious rooting in cucumber under salt stress. Meanwhile, we investigated that removal of ethylene suppressed calcium ion (Ca^2+^)-induced the formation of adventitious root under salt stress indicated that ethylene participates in this process. Moreover, the application of Ca^2+^ promoted the activities of 1-aminocyclopropane-l-carboxylic acid synthase (ACS) and ACC Oxidase (ACO), as well as the production of 1-aminocyclopropane-l-carboxylic acid (ACC) and ethylene under salt stress. Furthermore, we discovered that Ca^2+^ greatly up-regulated the expression level of *CsACS3*, *CsACO1* and *CsACO2* under salt stress. Meanwhile, Ca^2+^ significantly down-regulated *CsETR1*, *CsETR2*, *CsERS,* and *CsCTR1,* but positively up-regulated the expression of *CsEIN2* and *CsEIN3* under salt stress; however, the application of Ca^2+^ chelators or channel inhibitors could obviously reverse the effects of Ca^2+^ on the expression of the above genes. These results indicated that Ca^2+^ played a vital role in promoting the adventitious root development in cucumber under salt stress through regulating endogenous ethylene synthesis and activating the ethylene signal transduction pathway.

## 1. Introduction

Calcium ion (Ca^2+^), which is a versatile signaling ion, has been confirmed to play an essential role in plant growth [1,2] and development [3,4,5]. Moreover, Ca^2+^ could regulate the abiotic stress response in plants through various concentration of cytosolic free Ca^2+^. Recently, several researches have demonstrated that Ca^2+^ might play a positive role in regulating plant abiotic stresses [6,7]. For instance, Khan et al. [8] indicated that the drought stress-induced physiological damage in *Brassica* seedlings could be recovered via the application of Ca^2+^. In addition, several evidences have indicated that Ca^2+^ is involved in regulating the salt stress in plants. For instance, the exogenous Ca^2+^ application could partially protect seedlings under salt stress via declining sodium ion (Na+) and enhancing potassium ion (K+), Ca^2+^, and magnesium ion (Mg^2+^) in various plant organs of sour jujube seedlings [9]. Feng et al. [10] also found that FERONIA (FER)-mediated calcium signaling protected root cells through maintaining cell wall integrity under salt stress. As previous study reported, Ca^2+^ might be an extraordinary signaling molecule for inducing adventitious rooting under stress-free condition or stress condition, which interacts with other signaling molecules, such as nitric oxide (NO) [11,12], hydrogen peroxide (H_2_O_2_) [8,13], methane (CH_4_) [14], etc. For example, Niu et al. [12] found that Ca^2+^ promoted the NO-induced adventitious root formation of cucumber under simulated osmotic stress through enhancing the water retention, photosynthetic, and antioxidative activities. However, the mechanism of Ca^2+^ signaling transduction for affecting the development, growth, and abiotic stress response in plants needs further research.

Ethylene biosynthetic pathway has been extensively reported [15,16]. Previous reports have suggested that ethylene, as a vital plant hormone, which regulates a diverse array of physiological processes, including seed germination [17], root growth, and development [18,19,20], and shoot growth [21]. Furthermore, ethylene is a pivotal mediator in the response to biotic/abiotic stresses in plants [22,23]. It has been reported that the contribution of ethylene to salt acclimation processes can vary with respect to the type of response, including enhanced ethylene production and/or improved expression of ethylene receptors [24,25,26]. In addition, several lines of evidences indicate that ethylene might be involved in crosstalk with other signaling molecules during plant growth [27,28], development [29], and stress responses [30,31]. For example, the *SD1* gene (SEMIDWARF1), as a gibberellin biosynthesis gene that was transcriptionally activated by ethylene-responsive transcription factor OsEIL1a, is responsible for promoting internode elongation in deepwater rice [32]. Additionally, ethylene is demonstrated to be a downstream molecule of NO in affecting cell wall phosphorus reutilization of phosphorus-deficient rice [33].

It has been reported that Ca^2+^ and ethylene as signaling modulators are involved in the processes of plant growth and development, as well as stress response. For instance, Ferguson [34] found ethylene production that depends on 1-aminocyclopropane-l-carboxylic acid (ACC) and indole-3-acetic acid (IAA) pathways could be stimulated by Ca^2+^ in hypocotyls of mung bean and senescing cotyledons of cucumber, as well as in preclimacteric apple fruit. Hasenstein et al. [35] also find that Ca^2+^ could accelerate the conversion of ACC to ethylene in segments of primary roots of Zea mays. Cytosolic calcium is found to be responsible for gene expression of ethylene-induced ACC oxidase (*VR-ACO1*) in root tissue of mung bean [36]. They indicated that Ca2+ inhibitors ruthenium red, gadolinium chloride (GdCl_3_), and a calcium chelator significantly inhibited the gene expression of *VR-ACO1*, which was induced by ethylene, respectively [36]. Moreover, the application of Ca^2+^ enhanced the production of ethylene and decline abscisic acid (ABA) level in wheat seedlings to inhibit the growth and development of pathogen and decreased the degree of infection [37]. Li et al. [38] found that calcium-dependent protein kinase 5 (CPK5) and CPK6 were involved in the wounding-induced ethylene synthesis via the regulation of 1-aminocyclopropane-l-carboxylic acid synthase (ACS) genes expression. In addition, Zhao et al. [39] found that ethylene also activated a plasma membrane Ca^2+^-permeable channel to enhance the endogenous Ca^2+^ level in tobacco (*Nicotiana tabacum*) suspension cells. As mentioned above, the relationship between ethylene and Ca^2+^ might be an essential component in plant development, growth, and stress response. Salt stress is one of the major abiotic stresses in plants worldwide [40]. Previous studies have suggested that Ca^2+^ or ethylene may play a positive role in the development of adventitious root in plants under normal or stress conditions [12,18]. However, the crosstalk between Ca^2+^ and ethylene during adventitious rooting under abiotic stress is still unknown. It may be hypothesized that Ca^2+^, ethylene and their crosstalk may influence the adventitious root production under abiotic stress. Thus, in this study, some evidences have been provided regarding ethylene being involved in Ca^2+^-induced adventitious rooting under salt stress. Our results will help to improve our understanding the mechanism of Ca^2+^ signaling transduction in plants under abiotic stress.

## 2. Results

### 2.1. Effect of Exogenous Sodium Chloride (NaCl), Calcium and Ethylene on the Adventitious Rooting of Cucumber Explant under Salt Stress

**Figure 1:**
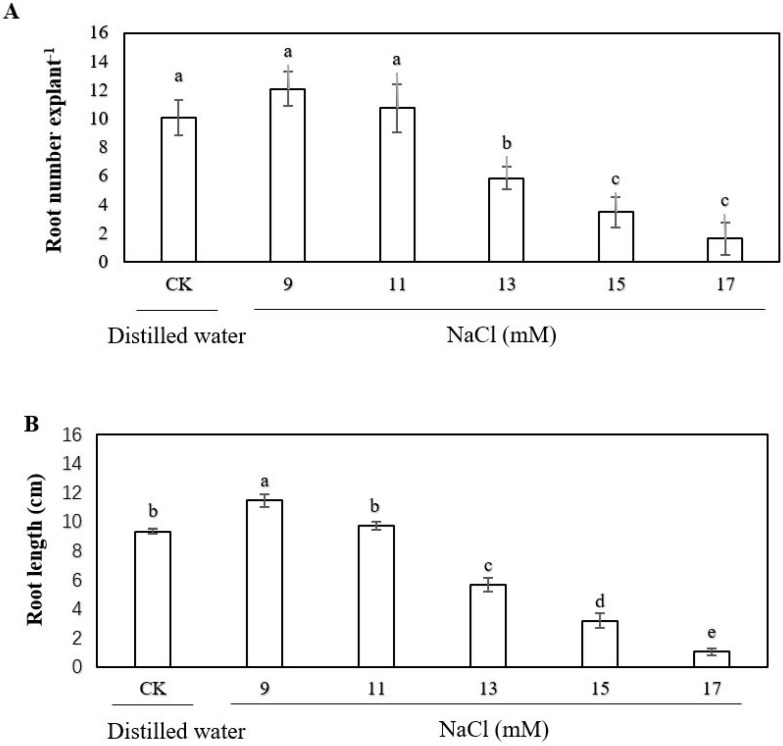
Effects of different concentrations of sodium chloride (NaCl) on adventitious root development in cucumber explants. The primary roots were removed from hypocotyl of five-day-old seedlings. The explants were incubated for three days with different concentration of NaCl after two days of distilled water culture. The numbers (**A**) and root length (**B**) of adventitious root were expressed as mean ± SE (n = 3, 10 explants were used per replicate). Bars with different lowercase letters were significantly different by Duncan’s multiple range test (*P* < 0.05).

In order to assess the effect of salt stress on adventitious rooting of cucumber, we performed a dose-response experiment with NaCl. When the cucumber seedlings were treated with different concentrations of salt, we found a significant effect on the formation of adventitious root of cucumber (Figure 1). As shown in (Figure 1A,B), the root number and root length under 13 mM NaCl decreased to about half that of the control treatment. Therefore, 13 mM NaCl was utilized to simulate salt stress in the following experiments.

**Figure 2:**
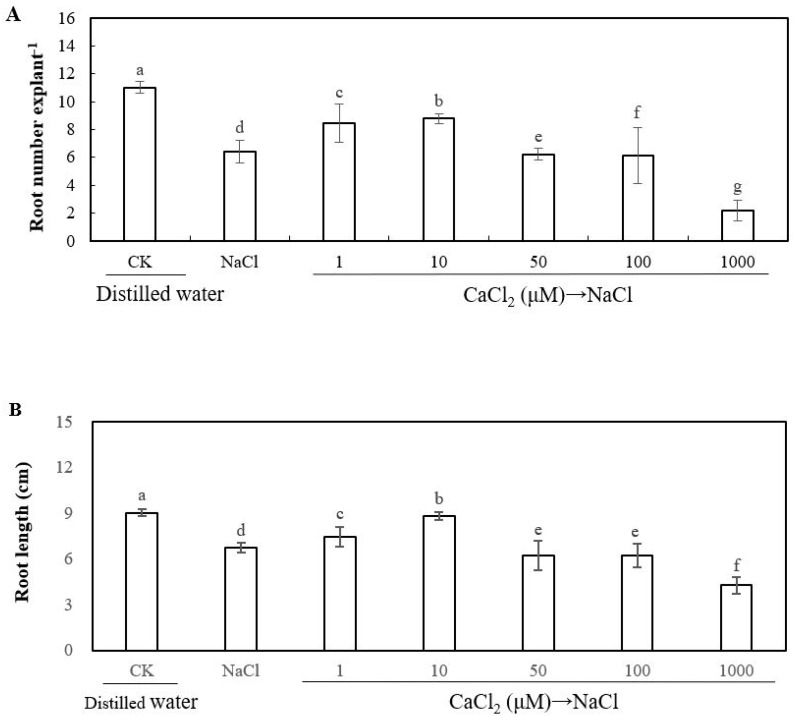
Effects of different concentrations of calcium chloride (CaCl_2_) on adventitious root development in cucumber explants under salt stress. The primary roots were removed from hypocotyl of five-day-old seedlings. The explants were incubated for five days with distilled water as control. Explants were incubated with 13 mM NaCl in three days after being treated with distilled water or different concentrations of CaCl_2_ treatments, respectively, for two days. The numbers (**A**) and root length (**B**) of adventitious root were expressed as mean ± SE (*n* = 3, 10 explants were used per replicate). Bars with different lowercase letters were significantly different by Duncan’s multiple range test (*P* < 0.05).

In order to investigate the effect of Ca^2+^ on the development of adventitious root under salt stress, cucumber explants were treated with different concentrations of calcium chloride (CaCl_2_). As shown in Figure 2, a lower concentration of CaCl_2_ (1 μM and 10 μM) treatment significantly increased the root number and root length under salt stress. However, higher concentrations of CaCl_2_ (50, 100, and 1000 μM) significantly decreased the root number and root length, which indicates that the effect of Ca^2+^ on root number and root length of adventitious roots was dose-dependent under salt stress. Additionally, the root number and root length of 10 μM CaCl_2_ treatment increased by 37.50% and 30.69%, respectively, when compared with those of NaCl treatment. These results indicate that 10 μM CaCl_2_ significantly reversed the adverse effect of salt stress and promoted the development of adventitious roots. Thus, 10 μM CaCl_2_ was utilized for further experiments to study the processes of adventitious rooting under salt stress.

**Figure 3:**
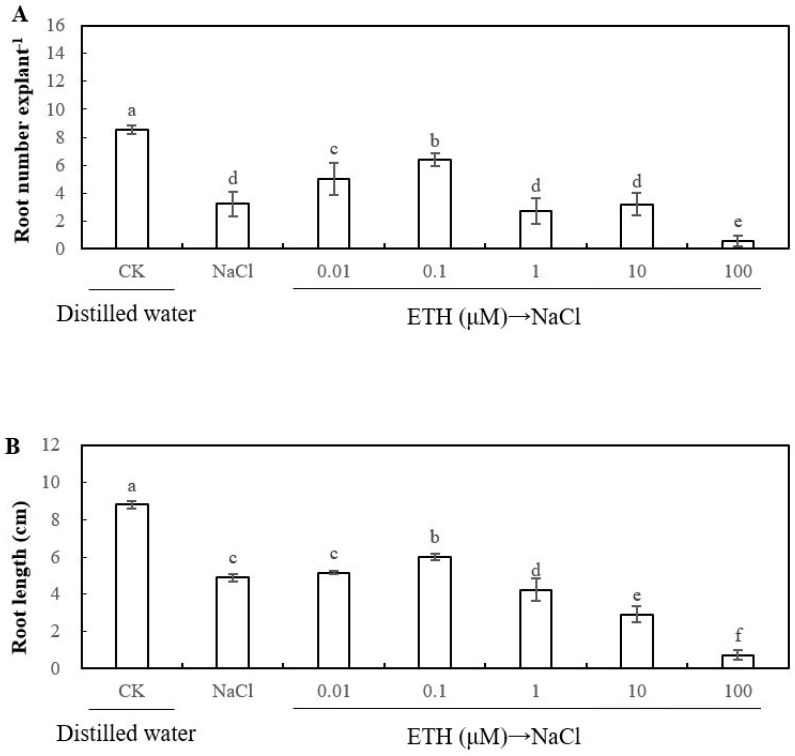
Effects of different concentrations of ethylene on adventitious root development in cucumber explants under salt stress. The primary roots were removed from hypocotyl of five-day-old seedlings. Explants were incubated for five days with distilled water as control. Explants were incubated with 13 mM NaCl in three days after being treated with distilled water or different concentrations of various concentration of ethylene donor 2-chloroethylphosphonic acid (ethrel) treatments for two days, respectively. The numbers (**A**) and root length (**B**) of adventitious root were expressed as mean ± SE (*n* = 3, 10 explants were used per replicate). Bars with different lowercase letters were significantly different by Duncan’s multiple range test (*P* < 0.05).

To evaluate the effect of ethylene on adventitious root development in cucumber explants under salt stress, we used different concentrations ethylene donor 2-chloroethylphosphonic acid (ethrel) to treat cucumber explants (Figure 3). There were marked increases in root number of cucumber explant that was treated with 0.01 μM or 0.1 μM ethrel under salt stress; it also existed in the root length of cucumber explant treated with 0.1 μM ethrel. However, higher concentrations of ethrel (1, 10, and 100 μM) significantly reduced the root number and root length under salt stress. In short, the effect of ethylene on root number and root length of adventitious root was dose-dependent under salt stress. Finally, since 0.1 μM ethrel achieved maximum biological effect during adventitious root formation under salt stress, we used 0.1 μM of ethylene for subsequent experiments.

### 2.2. Effect of Ethylene Aminoethoxyvinyl Glycine (AVG), Cobalt Chloride (CoCl_2_) and Sliver Nitrate (AgNO_3_) on the Adventitious Rooting of Cucumber Explant under Salt Stress

**Figure 4:**
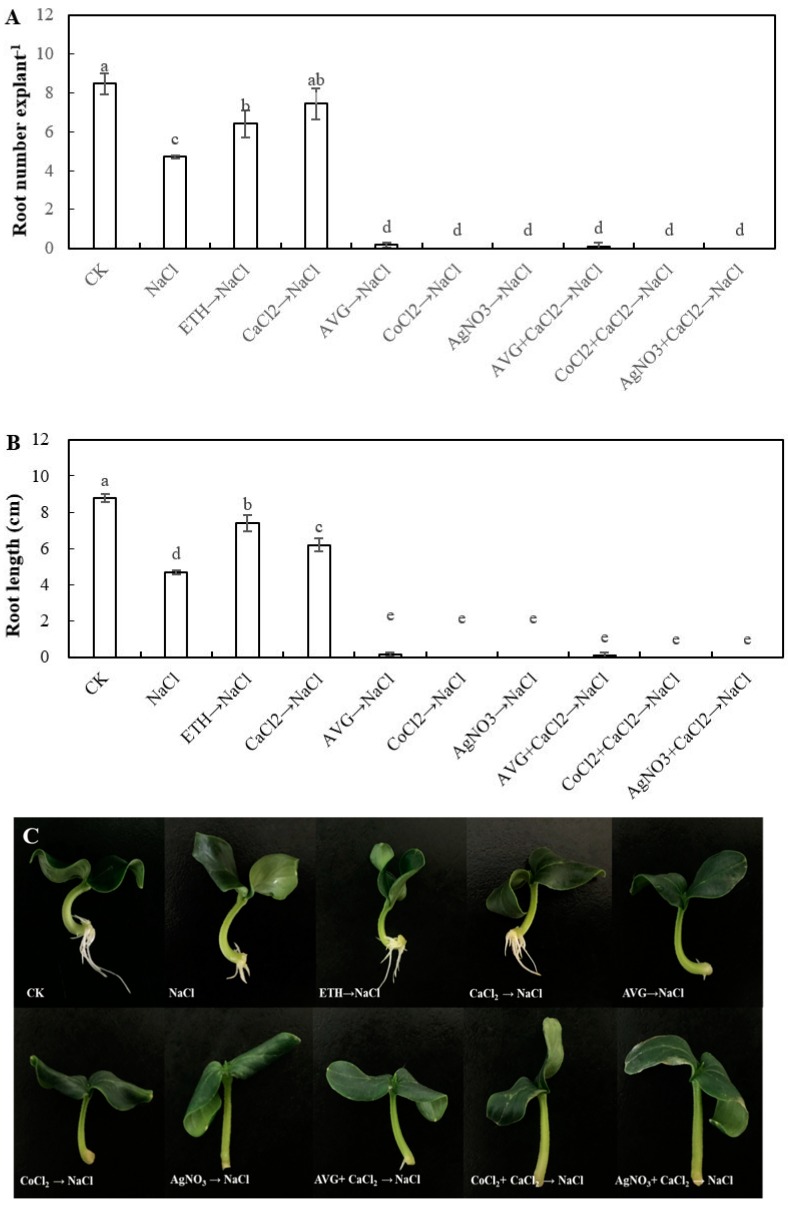
Effects of aminoethoxyvinyl glycine (AVG), cobalt chloride (CoCl_2_), and sliver nitrate (AgNO_3_) on adventitious root development in cucumber explants under salt stress. The primary roots were removed from hypocotyl of five-day-old seedlings. Explants were incubated for five days with distilled water as control. Explants were incubated with 13 mM NaCl in three days after being treated with distilled water or 0.1 μM ethrel, 10 μM CaCl_2_, 10 μM AVG, 20 μM CoCl_2_, 10 μM AgNO_3_, 10 μM AVG +10 μM CaCl_2_, 20 μM CoCl_2_ + 10 μM CaCl_2_, 10 μM AgNO_3_ +10 μM CaCl_2_ for two days, respectively. The numbers (**A**) and root length (**B**) of adventitious root were expressed as mean ± SE (*n* = 3, 10 explants were used per replicate). Bars with different lowercase letters were significantly different by Duncan’s multiple range test (*P* < 0.05). Photographs (**C**) show hypocotyl explants after five days of the treatments indicated.

In order to further investigate the requirement of ethylene for Ca^2+^-induced adventitious rooting in cucumber explant under salt stress, ethylene inhibitors, including AVG, CoCl_2_, and AgNO_3_ were applied in this study (Figure 4). The result showed that AVG, CoCl_2_, and AgNO_3_ significantly inhibited the development of adventitious roots under salt stress. In addition, AVG + CaCl_2_, CoCl_2_ + CaCl_2_, and AgNO_3_ + CaCl_2_ could not reverse the inhibition of adventitious rooting under salt stress.

### 2.3. Effect of Ca^2+^ on Ethylene Biosynthesis During Adventitious Rooting in Cucumber under Salt Stress

**Figure 5:**
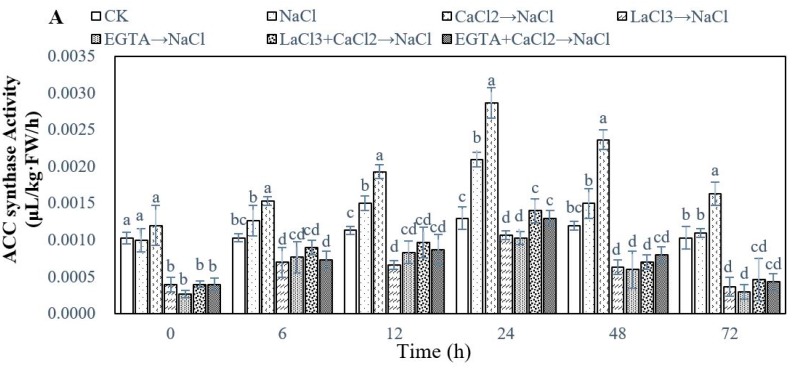

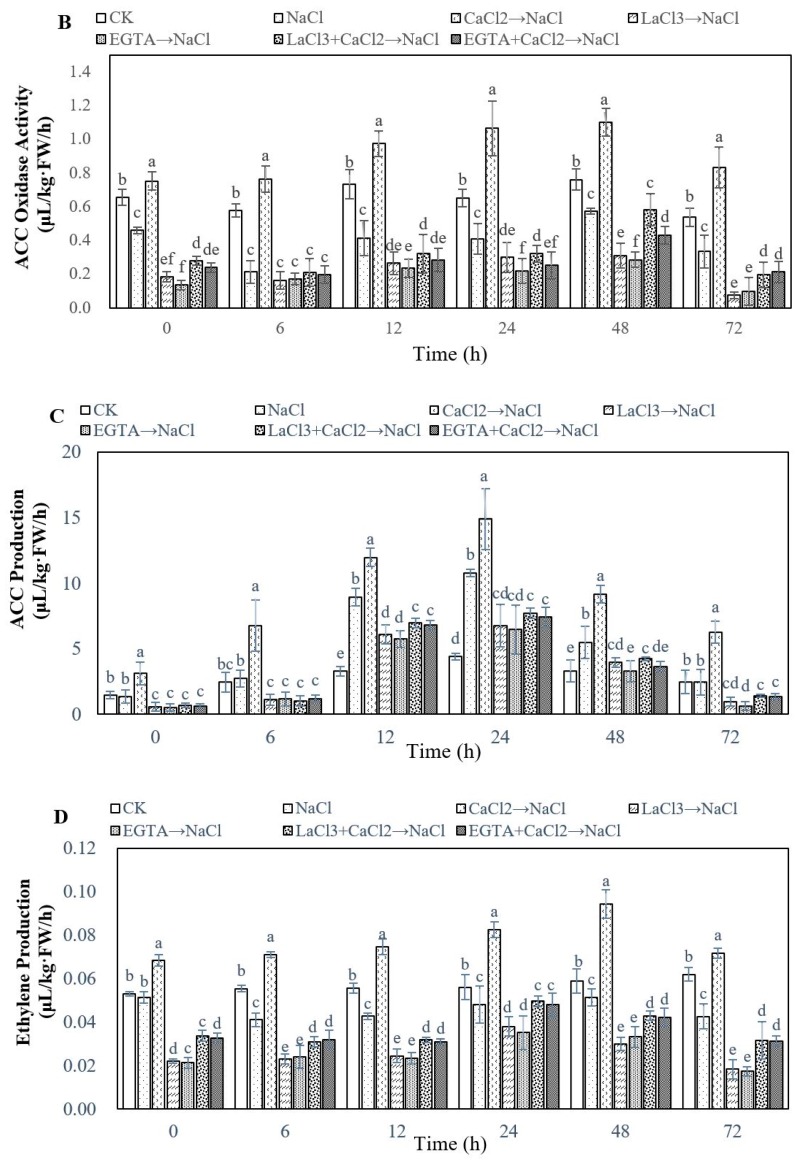
Effect of exogenous Ca^2+^ on 1-aminocyclopropane-l-carboxylic acid synthase (ACS) activity (**A**), ACC Oxidase (ACO) activity (**B**), 1-aminocyclopropane-1-carboxylic acid (ACC) production (**C**), and ethylene production (**D**) in cucumber explants under salt stress at different times. The primary roots were removed from hypocotyl of five-day-old seedlings. Explants were incubated for five days with distilled water as control, meanwhile explants were incubated with 13 mM NaCl after being treated with distilled water or 10 μM CaCl_2_, 500 μM lanthanum chloride (LaCl_3_), 100 μM methylene glycol-bis (2-aminoethylether) -N,N,N′,N′-tetraacetic acid (EGTA), 500 μM LaCl_3_ + 10 μM CaCl_2_, and 100 μM EGTA + 10 μM CaCl_2_, respectively. Values are expressed as means ± SE (*n* = 3), Bars with different lowercase letters were significantly different by Duncan’s multiple range test (*P* < 0.05).

To investigate whether exogenous Ca^2+^ will affect endogenous ethylene content on adventitious root development in cucumber explants under salt stress, we used the calcium channel inhibitor LaCl_3_ and calcium chelator methylene glycol-bis (2-aminoethylether) -N,N,N′,N′-tetraacetic acid (EGTA) to study their effect on ACS activity, ACO activity, ACC production, and ethylene production in different stages. The results showed that ACS activity (Figure 5A) in cucumber explant clearly increased up during 0–24 h and then decreased at 48 h and 72 h. All of the treatments reached their highest value at 24 h. For ACS activity at 24 h, NaCl significantly increased ACS activity as compared with CK, meanwhile CaCl_2_ markedly enhanced ACC activity under salt stress. While, LaCl_3_ + CaCl_2_ and EGTA + CaCl_2_ could noticeably increase ACS activity when compared with LaCl_3_ and EGTA significantly reduced ACS activity in cucumber explant under salt stress. As shown in Figure 5B, the activity of ACO increased first and then decreased with time, and all of the treatments reached their maximum value at 48 h. NaCl treatment significantly decreased the ACO activity, nevertheless CaCl_2_ could remarkably increase ACO activity under salt stress. Meanwhile, LaCl_3_ and EGTA marked decrease ACO activity, but LaCl_3_ + CaCl_2_ and EGTA + CaCl_2_ could alleviate the decrease of ACO activity under salt stress. A change of ACC production (Figure 5C) coincided with ACS activity, while ethylene production (Figure 5D) and ACO activity had a similar trend.

### 2.4. Effects of Ca^2+^ on the Expression Levels of CsACS1, CsACS2, CsACS3, CsACO1and CsACO2 under Salt Stress During Adventitious Rooting in Cucumber

**Figure 6:**
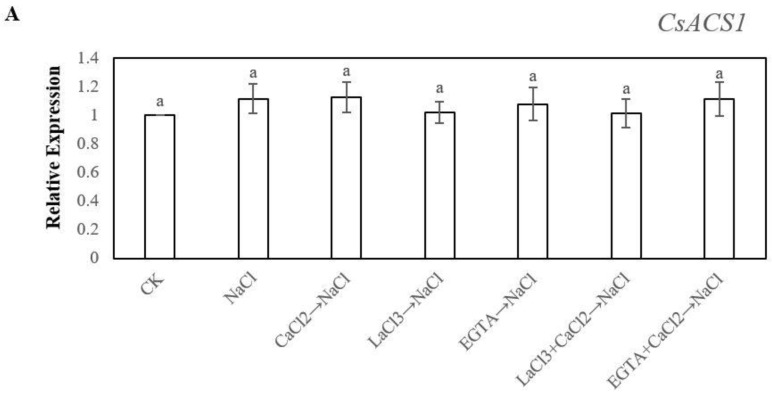

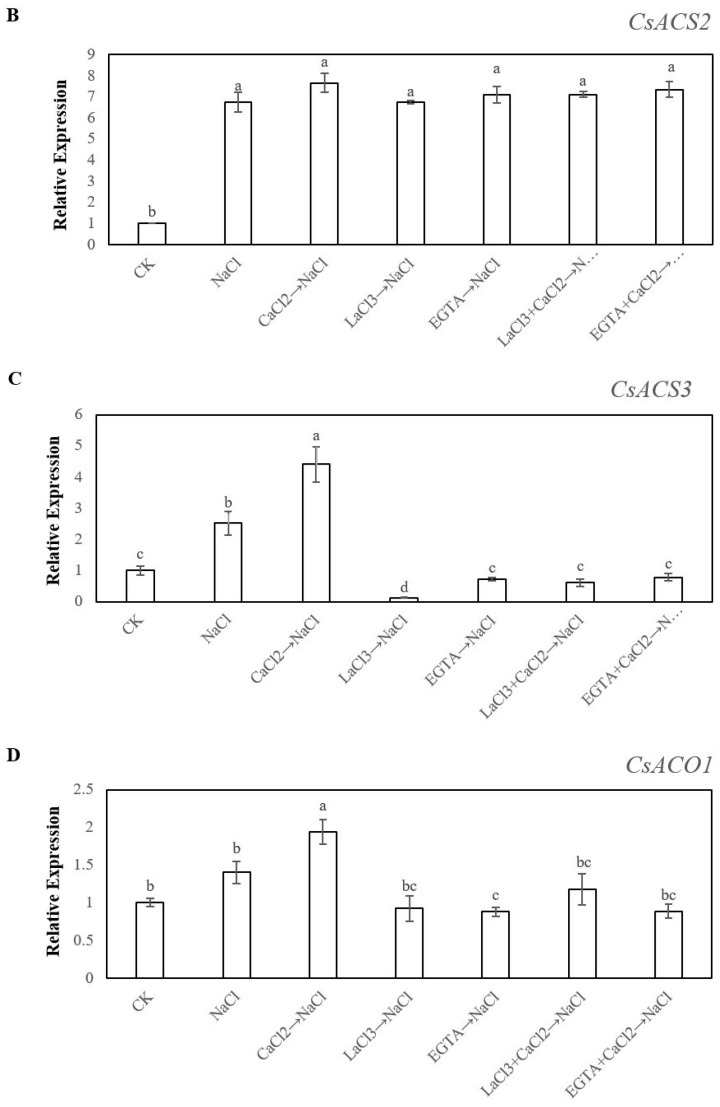

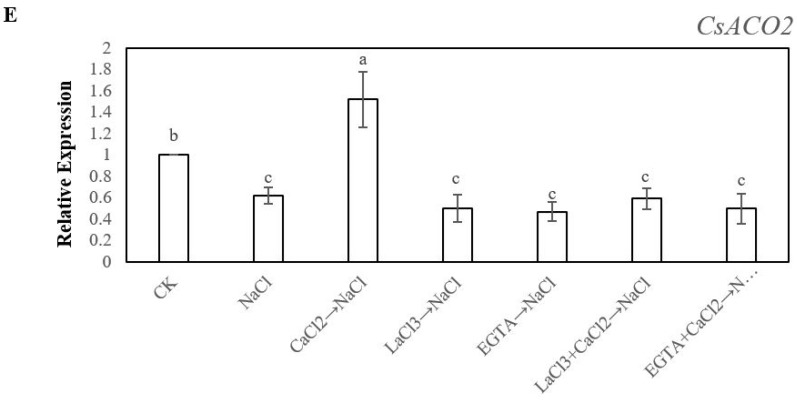
Effects of Ca^2+^ on the Expression Levels of ACS gene *CsACS1* (**A**), *CsACS2* (**B**), *CsACS3* (**C**), and ACO gene *CsACO1* (**D**), *CsACO2* (**E**) in cucumber explant under salt stress at 48 h. The primary roots were removed from hypocotyl of 5-day-old seedlings. Explants were incubated for five days with distilled water as control, meanwhile the explants were incubated with 13 mM NaCl after being treated with distilled water or 10 μM CaCl_2_, 500 μM LaCl_3_, 100 μM EGTA, 500 μM LaCl_3_ + 10 μM CaCl_2_, and 100 μM EGTA + 10 μM CaCl_2_, respectively. The values (means ± SE) are the average of three independent experiments. Bars with different lowercase letters were significantly different by Duncan’s multiple range test (*P* < 0.05).

Because ethylene production reached its highest value at 48 h, in order to investigate whether exogenous Ca^2+^ could affect endogenous ethylene content on adventitious root development in cucumber explants under salt stress, we determined the expression of the genes encoding ACS and ACO. The result suggested that the expressions of *CsACS1* in all of the treatments have significant difference at 48 h (Figure 6A). All of the treatments under salt stress significantly up-regulated the expression of *CsACS2* when compared with control. However, there was no significant difference among the treatments (Figure 6B). As shown in Figure 6C, NaCl treatment marked up-regulated the expression of *CsACS3*, meanwhile, exogenous CaCl_2_ remarkable up-regulated *CsACS3* expression under salt stress. Nevertheless, LaCl_3_, EGTA, LaCl_3_ + CaCl_2_, and EGTA + CaCl_2_ significantly down-regulated the expression of *CsACS3* under salt stress. For the ACO gene (Figure 6D,E), NaCl treatment significantly up-regulated the expression of *CsACO1* when compared with control, but down-regulated the expression of *CsACO2*. Exogenous CaCl_2_ markedly up-regulated the expression of *CsACO1* and *CsACO2* in cucumber explant under salt stress, but LaCl_3_, EGTA, LaCl_3_ + CaCl_2_, and EGTA + CaCl_2_ treatments all down-regulated the expression of *CsACO1* and *CsACO2* under salt stress.

### 2.5. Effects of Ca^2+^ on the Expression Levels of CsETR1, CsETR2, CsERS, CsCTR1, CsEIN2 and CsEIN3 under Salt Stress During Adventitious Rooting in Cucumber

**Figure 7:**
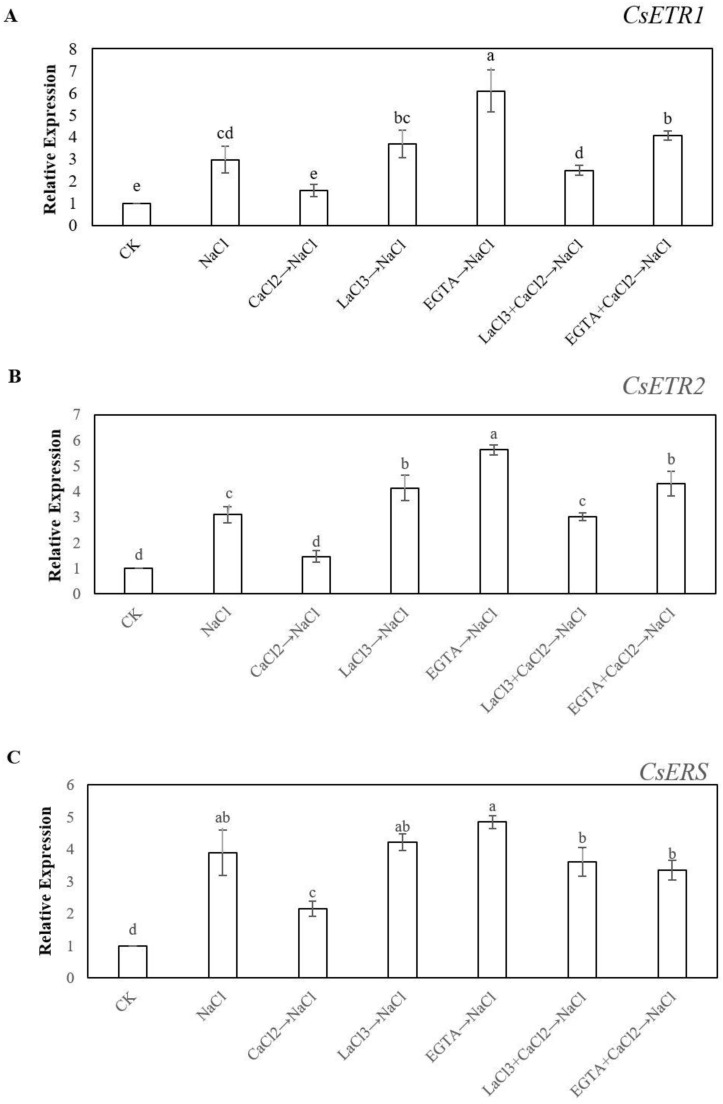

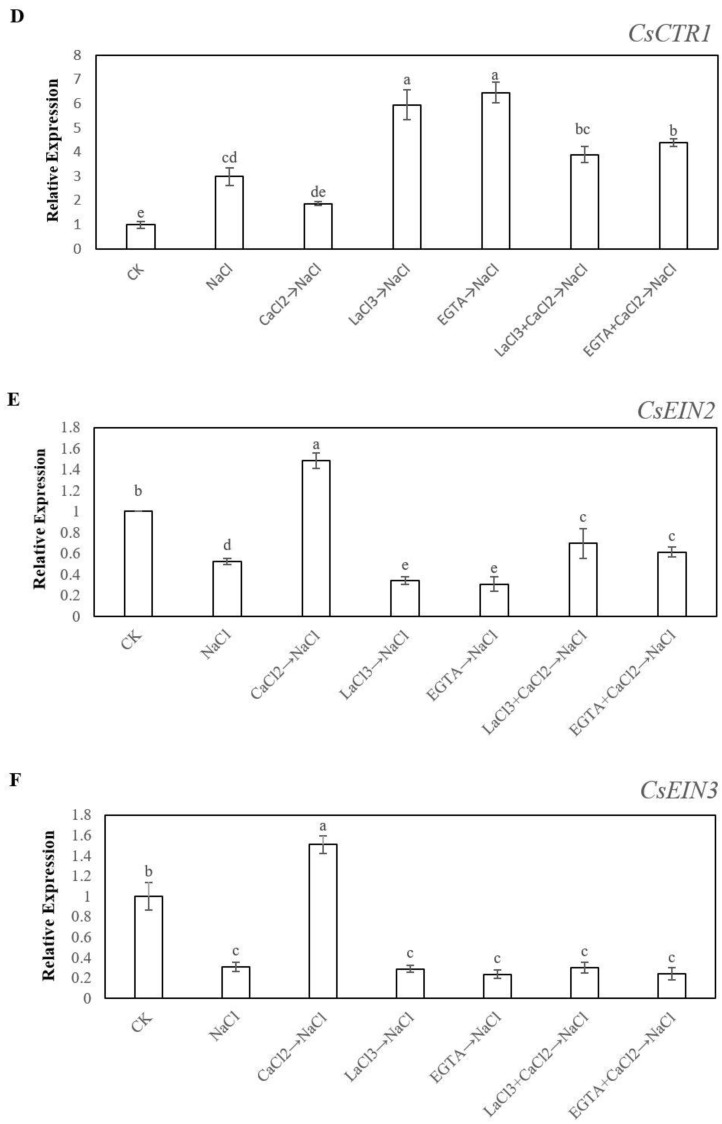
Effects of Ca^2+^ on the Expression Levels of *CsETR1* (**A**), *CsETR2* (**B**), *CsERS* (**C**), *CsCTR1* (**D**), *CsEIN2* (**E**), and *CsEIN3* (**F**) in cucumber explant under salt stress at 48 h. The primary roots were removed from hypocotyl of five-day-old seedlings. Explants were incubated for five days with distilled water as control, meanwhile the explants were incubated with 13 mM NaCl after being treated with distilled water or 10 μM CaCl_2_, 500 μM LaCl_3_, 100 μM EGTA, 500 μM LaCl_3_ + 10 μM CaCl_2_, 100 μM EGTA + 10 μM CaCl_2_, respectively. The values (means ± SE) are the average of three independent experiments. Bars with different lowercase letters were significantly different by Duncan’s multiple range test (*P* < 0.05).

We evaluated the effect of Ca^2+^ on the signal perception of ethylene on adventitious root development in cucumber explants under salt stress. As shown in Figure 7A, the expression of *CsETR1*, which encoded the ethylene receptor, was significantly higher under NaCl treatment when compared with the control. However, CaCl_2_ treatment under salt stress significantly decreased the expression of *CsETR1*. In addition, LaCl_3_ and EGTA remarkably up-regulated the expression of *CsETR1* under salt stress. Nevertheless, the LaCl_3_ + CaCl_2_ and EGTA + CaCl_2_ treatments markedly retarded the expression of *CsETR1* as compared with LaCl_3_ and EGTA, respectively. The other two ethylene receptor genes, *CsETR2* and *CsERS,* had a similar trend in gene expression with *CsETR1* (Figure 7B,C). We also investigated the expression of *CsCTR1*, a cucumber CTR-like gene, which has a semblable expression level with ethylene receptor genes (Figure 7D). In addition, we tested the expression of *CsEIN2* and *CsEIN3*. From the Figure 7E,F, NaCl treatment significantly reduced the expression of *CsEIN2* and *CsEIN3*. However, exogenous CaCl_2_ enhanced the expression of *CsEIN2* and *CsEIN3* under salt stress. Moreover, LaCl_3_ and EGTA could significantly decrease the expression of *CsEIN2* and *CsEIN3* under salt stress, while LaCl_3_ + CaCl_2_ and EGTA + CaCl_2_ markedly up-regulated the expression of *CsEIN2* as compared with LaCl_3_ and EGTA, but for *CsEIN3* under salt stress.

## 3. Discussion

Previous researches have suggested that Ca^2+^ or ethylene might play important roles in responding to abiotic stress [12,21,41]. Moreover, several researches have shown that salt stress has a negative effect on the formation of a root system, such as primary root, lateral root, and root hair [42,43,44]. According to previous report, there is little research regarding the relationship between calcium and ethylene on adventitious root formation under salt stress. In this study, our results confirmed that ethylene is involved in Ca^2+^-induced adventitious rooting of cucumber under salt stress. 

As previously described, Ca^2+^ has been confirmed to play a key role in the process of plant growth and development, such as seed germination [1], root growth and development [5,11,45], and other physiological processes [3,46]. Additionally, Ca^2+^ is involved in resisting abiotic stress in plants [47,48]. As shown in Figure 2, a suitable dose of Ca^2+^ significantly increased the adventitious root number and length under salt stress, which is higher than that of NaCl treatment. Besides, some researches demonstrated that the concentration of CaCl_2_ in μM range significantly promoted adventitious rooting, which implied that Ca included in the μM range could have an obvious effect on regulating adventitious root development [11,49] Previously, Ca^2+^ has been demonstrated to induce the adventitious rooting of cucumber under abiotic stress. For instance, Niu et al. [12] also found that Ca^2+^ played a positive role in the NO-induced adventitious root development under osmotic condition by enhancing water retention, photosynthetic activity, and antioxidant response. Our results are consistent with those of Niu et al. [12], which showed that Ca^2+^ could regulate adventitious root formation in cucumber under abiotic stress. Cramer et al. [50] suggested that a positive curvilinear relationship exists between root growth and the Ca^2+^/Na^+^ ratio in the nutrient solution. However, these results are different from our research’s results (Figure 2). The reason for the different results might probably be because the explants in our experiment were cultivated in a different concentration of Ca^2+^ solution for two days and then transferred to NaCl solution alone for three days, namely the possible interaction effect between Ca^2+^ and Na^+^ could be negligible in two discrete solutions during our experiment. In addition, ethylene has also been found to exhibit a positive role in enhancing adventitious rooting [18,19,51,52]. For instance, ethylene promoted adventitious rooting through activating endogenous NO synthesis and improving the expression of related genes that are responsible for adventitious rooting, such as *CsDNAJ-1* and *CsCDPK1/5* [18]. Moreover, ethylene plays an essential role in plant response to abiotic stress through regulating abiotic stress-responsive gene expression [41]. According to our results, we found that ethylene has a promotive effect on adventitious rooting under salt stress (Figure 3). However, a study showed that ethylene might be an inhibitor of adventitious rooting in tomato leaf discs [53]. The reasons for the different results of ethylene affects adventitious rooting may be due to variation in tissue differentiation, growth conditions, and the quantification standard of adventitious root formation [54]. In the subsequent experiment, ethylene inhibitors were utilized in order to investigate whether ethylene was involved in Ca^2+^-induced adventitious rooting under salt stress. Our results indicated that the application of AVG, CoCl_2_, or AgNO_3_ significantly suppressed Ca^2+^-induced adventitious rooting under salt stress (Figure 4). These results implied that ethylene might be as a downstream signaling molecule of Ca^2+^ to induce the formation of adventitious rooting under salt stress.

Crosstalk with Ca^2+^ and ethylene signaling has been found in plants. For example, the CPK pathway modulated wounding-induced ethylene production through regulating the expression of *ACS* genes [38]. Besides, Jin et al. [55] demonstrated that CPK28 could degrade methionine adenosyltransferase (MAT) by the 26S proteasome and thus affect endogenous ethylene biosynthesis in *Arabidopsis*. Additionally, Ludwig et al. [56] indicated that ethylene is involved in crosslinking between CPK and MAPK signaling controls stress responses in plants. Our results showed that the application of Ca^2+^ significantly increased the endogenous ethylene synthesis through enhancing the activity and related gene expression of ACS and ACO, as well as the ACC level in cucumber hypocotyls during adventitious rooting under salt stress (Figure 5 and Figure 6). Previously, Burns and Evensen [57] found that Ca^2+^ could increase endogenous ethylene content through increasing the ACS activity from ripening tomato. Moreover, Ca^2+^ might promote ACC formation for conversion to ethylene [35,58]. The expression of active *Nicotiana tabaccum* CDPK gene (*NtCDPK2)* had a higher increase in the ACC level of the transgenic plant [56]. Also, CDPKs, AtCPK4, and AtCPK11, which are activated by ABA, are proven to phosphorylate *ACS6* for producing more endogenous ethylene [59]. These results indicated that Ca^2+^ signaling indeed regulates the ethylene synthesis pathway in plant cell and there exists a close relationship between Ca^2+^ and ethylene in plants. In our study, we further found that Ca^2+^ might induce adventitious root development via enhancing the endogenous ethylene level under salt stress. Additionally, a previous study demonstrated that Ca^2+^ depletion via pre-treatment with EGTA, verapamil, and LaCl_3_ all inhibit the ethylene response by suppressing the activity and transcriptional level of ACO enzyme [60]. Furthermore, calcium signaling blockers, EGTA, or LaCl_3_ significantly suppressed the ripening and gene expression of *MaCDPK7*, *MaACO1*, and *MaACS1* in vitro cultured peeled pieces of ripe banana. However, the application of Ca^2+^ remarkably removed the inhibitory effect of EGTA and LaCl_3_ [61]. Our results suggested that EGTA or LaCl_3_ significantly decreased the production of endogenous ethylene in hypocotyl by inhibiting ACC content, the activities of ACS and ACO enzyme, and the expression of *ACS2*, *ACS3*, *ACO1,* and *ACO2* genes (Figure 5 and Figure 6). Wang et al. [62] also found that *ACO1* and *ACO2* might be required for the increase in ACO-mediated ethylene synthesis under salt stress. Therefore, these results suggest that the activation of endogenous ethylene synthesis pathways is required for the promotive effect of Ca^2+^-induced adventitious rooting under salt stress. Interestingly, in our study, we found that the presence or absence of Ca^2+^ has no effect on the expression level of *ACS1* (Figure 6). A previous study has found that EBR decreased the expression of *ACS2*, *ACS3* genes and had no influence on *ACS1* expression during seed germination under salt stress [62] and the different results of gene expression were probably because of the different growth conditions and different stage of growth and development in cucumber.

**Figure 8:**
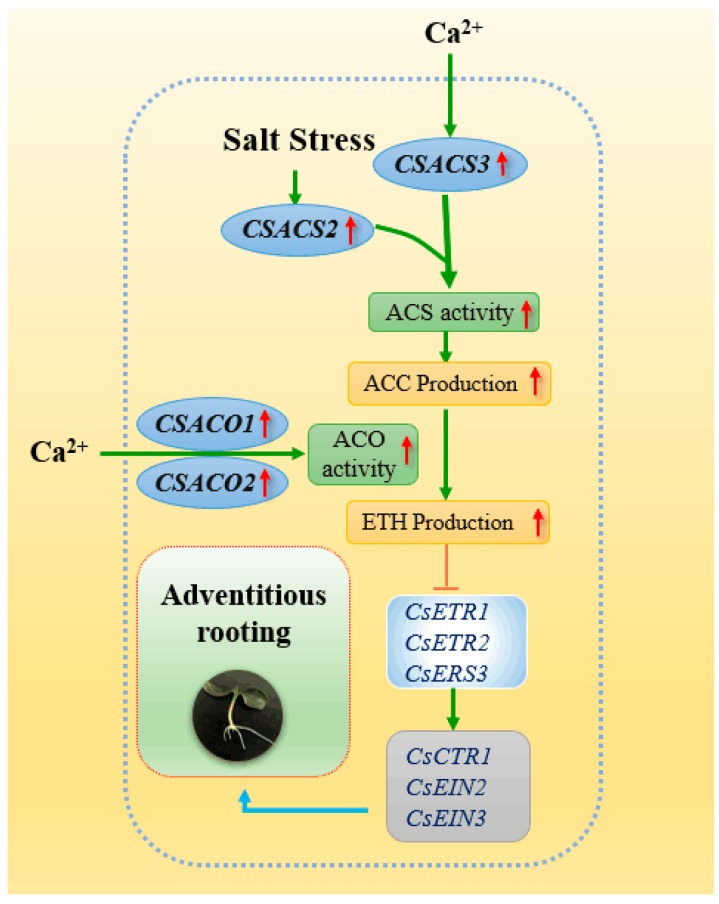
Schematic model of the signaling networks involving Ca^2+^ and ethylene during adventitious rooting in cucumber under salt stress. Ca^2+^ could promote the development of adventitious root under salt stress through regulating the activities and transcriptional levels of ACS and ACO enzyme to trigger endogenous ethylene accumulation. Meanwhile, the receptors and downstream components of ethylene signaling pathway could be regulated by Ca^2+^ during adventitious rooting under salt stress. T bars, inhibition.

Previous studies suggest that ethylene triggers a series of signaling cascades that are initiated by ethylene receptors, which includes *ERS1*, *ERS2*, *ETR1*, *ETR2*, *EIN4,* and *CTR1* protein, which are negative regulators of the ethylene response [21,63]. However, *EIN2* and *EIN3* are the positive regulators of the ethylene response [64]. In another experiment, our results demonstrated that Ca^2+^ might significantly retard the expression of *ETR1*, *ETR2*, *ERS,* and *CTR1* genes, and up-regulated the *EIN2* and *EIN3* expression level (Figure 7). Previous studies have shown that ethylene receptors might regulate plant growth and development, such as seed germination [24], fruit development [65], and response to abiotic stress [25]. Duckett et al. [66] found that ctr1 mutation resulted in longer hairs than those of the wild type, which implied that CTR1 might also be involved in the formation of root hair. Moreover, previous research indicated *ein2* mutant has an abnormality in root hair elongation [67]. Our results demonstrated that Ca^2+^ could regulate these ethylene receptors for adventitious root production. However, the application of EGTA or LaCl_3_ remarkably reversed the positive effect of Ca^2+^ (Figure 7), implying that the ethylene receptors are involved in Ca^2+^-promoted adventitious root formation under salt stress. Interestingly, Pitts et al. [67] also found that etr1 has a negative effect for root hair elongation in *Arabidopsis*; these results are different from our research’s results (Figure 7), which might be due to the different systems of root development, such as primary root, adventitious root, and so on. Therefore, considerably more work will be focus on the mechanism of Ca^2+^ and ethylene signaling transduction during adventitious rooting under the stress-free or stress condition. Our results have shown that the application of Ca^2+^ and ethylene could reverse the adverse effects of NaCl stress during adventitious rooting and ethylene might be downstream molecules of the Ca^2+^ signaling pathway (Figure 8). However, the interaction between Ca^2+^ and ethylene for adventitious rooting is complex. Therefore, considerably more work will be focus on the mechanism of Ca^2+^ and ethylene signaling transduction during adventitious rooting under the stress-free or stress condition.

## 4. Materials and Methods

### 4.1. Plant Materials

Cucumber (*Cucumis sativus* ‘Xinchun 4’, Gansu Academy of Agricultural Sciences, Lanzhou, China) seeds were germinated in petri dishes on filter papers that were moistened with distilled water and maintained at 25 °C with a 14 h photoperiod (photosynthetically active radiation = 200 μmol s^−1^ m^−2^) for five days in a climate box. Primary roots of five days old seedlings were removed and the cucumber explants were then maintained under the same conditions of temperature and photoperiod for another five days in the presence of different media, as indicated below. These media were changed every day in order keep the solution fresh.

### 4.2. Treatments of Explants

Experiment 1: sodium chloride (NaCl, Solarbio, Beijing, China) was used to simulate salt stress. Calcium chloride (CaCl_2_, Solarbio, Beijing, China) and 2-Chloroethylphosphonic acid (ethrel, Sigma, St Louis, MO, USA) as donor of Ca^2+^ and ethylene, respectively. We used different concentrations of NaCl (9, 11, 13, 15, and 17 mM), CaCl_2_ (1, 10, 50, 100, and 1000 μM), and ethrel (0.01, 0.1, 1, 10, and 100 μM), as indicated in Figure 1, Figure 2, and Figure 3 and kept at 25 °C. Aminoethoxyvinyglycine (AVG, Sigma, St Louis, MO, USA), cobalt chloride (CoCl_2_, Solarbio, Beijing, China), and sliver nitrate (AgNO_3_, Solarbio, Beijing, China) were used at 10, 20, and 10 μM, respectively, as ethylene inhibitor. The experiment included 10 treatments: (1) CK (the control), explants that were treated with distilled water for five days. (2) Distilled water → NaCl (NaCl treatment), explants pretreated with distilled water for two days, and then transferred to NaCl solution for three days. (3) ethylene → NaCl (ETH treatment), explants pretreated with ethrel for two days and then transferred to NaCl solution for three days. (4) CaCl_2_ → NaCl (CaCl_2_ treatment), explants pretreated with CaCl_2_ for two days, and then transferred to NaCl solution for three days. (5) AVG → NaCl (AVG treatment), explants pretreated with AVG for two days and then transferred to NaCl solution for three days. (6) CoCl_2_ → NaCl (CoCl_2_ treatment), explants pretreated with CoCl_2_ for two days, and then transferred to NaCl solution for three days. (7) AgNO_3_ → NaCl (AgNO_3_ treatment), explants pretreated with AgNO_3_ for two days, and then transferred to NaCl solution for three days. (8) AVG + CaCl_2_ → NaCl (AVG + CaCl_2_ treatment), explants pretreated with AVG + CaCl_2_ for two days, and then transferred to NaCl solution for three days. (9) CoCl_2_ + CaCl_2_ → NaCl (CoCl_2_ + CaCl_2_ treatment), explants pretreated with CoCl_2_ + CaCl_2_ for two days, and then transferred to NaCl solution for three days. (10) AgNO_3_ + CaCl_2_ → NaCl (AgNO_3_ + CaCl_2_ treatment), explants pretreated with AgNO_3_ + CaCl_2_ for two days, and then transferred to NaCl solution for three days.

Experiment 2: We used 100 μM Methylene glycol-bis (2-aminoethylether) -N,N,N′,N′-tetraacetic acid (EGTA, Sigma, St Louis, MO, USA) and 500 μM lanthanum chloride (LaCl_3_, Solarbio, Beijing, China) as Ca chelators and channel inhibitors, respectively. This experiment had seven treatments: (1) CK (the control), explants treated with distilled water for five days. (2) Distilled water → NaCl (NaCl treatment), explants pretreated with distilled water for two days and then transferred to NaCl solution for three days. (3) CaCl_2_ → NaCl (CaCl_2_ treatment), explants pretreated with CaCl_2_ for two days, and then transferred to NaCl solution for three days. (4) LaCl_3_ → NaCl (LaCl_3_ treatment), explants pretreated with LaCl_3_ for two days, and then transferred to NaCl solution for three days. (5) EGTA → NaCl (EGTA treatment), explants pretreated with EGTA for two days, and then transferred to NaCl solution for three days. (5) EGTA → NaCl (EGTA treatment), explants pretreated with EGTA for two days, and then transferred to NaCl solution for three days. (6) CaCl_2_ + LaCl_3_ → NaCl (CaCl_2_ + LaCl_3_ treatment), explants pretreated with CaCl_2_ + LaCl_3_ for two days, and then transferred to NaCl solution for three days. (7) CaCl_2_ + EGTA → NaCl (CaCl_2_ + EGTA treatment), explants pretreated with CaCl_2_ + EGTA for two days, and then transferred to NaCl solution for 3 days.

### 4.3. Determination of ACC Production

ACC production in explant was investigated using a modified procedure of Concepcion et al. [68]. Cucumber explants (0.5 g) were ground with 5 mL 80% ethanol and then incubated at 4 °C for 12 h. Solutions were dissolved in 2 mL chloroform and 4 mL ddH_2_O, then centrifugated 5 min under 4 ℃ and 4000 rpm, and transferred 0.5 mL upper later solution into 7 mL vial. Finally, adding 40 μL of 50 mM mercuric chloride (HgCl_2_) and 0.5 mL of mixed solution consisting 5% sodium hypochlorite (NaClO) and saturated sodium hydroxide (NaOH) (*V/V* = 2:1), subsequently stoppered with secure rubber caps. After incubation in ice-bath for 2 h, 1 mL headspace gas was taken from the vial, and injected into the gas chromatographic analyzer (Agilent 7820A, Santa Clara, CA, USA) that was equipped with a flame ionization detector (Agilent, Santa Clara, CA, USA).

### 4.4. Ethylene Measurement

In order to measure ethylene, three fresh cucumber explants were placed into 7 mL vial containing moist paper and stoppered with secure rubber caps, placed in light for 12 h under same condition, and then 1 mL headspace gas from the vial injected into the gas chromatographic analyzer.

### 4.5. Determination of ACO Activity

To examine the ACO activity, we used the procedure of Liu et al. [69], the 0.5 g quantity of explant was ground in liquid nitrogen with 5 mL of the extract mixture (0.1 M Tris-HCl, pH 7.2, 5% (*w/v*) Polyvinyl Pyrrolidone (PVP), 10% (*v/v*) glycerol, and 30 mM sodium ascorbate), followed by centrifugation at 15,000× *g* for 20 min at 4 °C. A total of 1 mL of clear supernatant was transferred into 7 mL vial and then added to 1.7 mL of extraction buffer (without polyvingypyrrolidone (PVPP)) containing 50 mM ferrous sulfate (FeSO_4_) and 2 mM ACC, subsequently stoppered with secure rubber caps, and then incubated at 30 °C for 1 h. Finally, 1 mL headspace gas was taken from the vial and injected into the gas chromatographic analyzer.

### 4.6. Determination of ACS Activity

In order to determine the activity of ACS, we used the test method of Liu et al. [69], 0.5 g of explants was finely ground in liquid nitrogen with the extract mixture (200 mM phosphate buffer, pH 8.0, 5 mM dithiothreitol, 2 mm phenylmethylsulfonyl fluoride, 1 mM ethylene diamine tetraacetic acid (EDTA) and 10 μM pyridoxal phosphate), and then centrifugation was undertaken for 15,000× *g* for 20 min at 4 °C. Pipette 1.5 mL supernatant to 7 mL of vial containing 0.5 mL 5 mM S-(50-adenosyl)-L-methionine. Afterwards, react for 1 h at 22 °C and add 0.2 mL of 1: 1 (*v/v*) NaOH: bleach and 100 mM HgCl_2_ into the vial. The vials were kept in ice for 20 min and 1 mL headspace gas was taken from the vial and then injected into the gas chromatographic analyzer.

### 4.7. Gene Expression Analyses by RT-qPCR

The method of Quantitative Real-time PCR (qRT-PCR) analyses and consequent statistical data analyses reference the procedure of Zhao et al. [70]. The cDNA was amplified using the following primers shown in Table 1. The expression analyses were independently conducted three times.

### 4.8. Statistical Analysis

The results were expressed as the mean values ± SE and each treatment include three independent replicates. Data was analyzed with the Statistical Package for Social Sciences for Windows (Version 13.00; SPSS, Inc, Chicago, IC, United States). Analysis of Variance (ANOVA) was done and statistical differences among the treatments were analyzed through Duncan’s multiple range test (*P* < 0.05).

## Figures and Tables

**Table 1:** Primers used for Quantitative Real-time PCR (qPT-PCR) assays.

Gene Symbol	Accession Number	Forward Primer (5′-3′)	Reverse Primer (5′-3′)	Primer Efficiency
Actin	AB010922.1	5′-TTGAATCCCAAGGCGAATAG-3′	5′-TGCGACCACTGGCATAAAG-3′	1.98
*CsACS1*	AB006803.1	5′-ACGGTCACGGCGAGGATTCAC-3′	5′-AGTTCAGGAGACCTTCGTCGGTAC-3′	1.96
*CsACS2*	AB006804.1	5′-ATGTCACCACACTCACCGTTGC-3′	5′-ATGACTGATTGGCGGTCGTCTTG-3′	1.99
*CsACS3*	AB006805.1	5′-CCTTGCAGAGGCTGGCGATG-3′	5′-GGTGACTTGGAAGCCGTTGGAG-3′	1.99
*CsACO1*	AB006806.1	5′-AGGTAGGTGGCCTGCAACTCC-3′	5′-CTCCGAGGTTGACGACAATGGC-3′	1.99
*CsACO2*	AB006807.2	5′-CAGTCTCCAACATCGCGGATCTC-3′	5′-GCAGGAGTTCGGCGAGTACTTG-3′	1.92
*CSETR1*	AB026498.1	5′-AATGAGGAGCGTGTTGTCGGAAC-3′	5′-TCTCAAGATCACCACCACAATGCC-3′	1.96
*‘CSETR2*	NM_001308840.1	5′-GCCATGCCTGAACCTGGAGAATC-3′	5′-GCTGGTGCCATGACTGTGAGAC-3′	1.97
*CSERS*	AB026499.1	5′-AGAAGTTGTTGCAGTGCGAGTCC-3′	5′-GCTACCTGGTCTGCGACAACATC-3′	1.95
*CsCTR1*	NM_001305781.1	5′-TGTTGACTCCAGCATCGCTTCATC-3′	5′-CAAGTGATTGCATACCAGCTTCGC-3′	1.97
*CsEIN2*	NM_KF245636.1	5′-ATTATCAGCCTGCCACAGTCCATG-3′	5′-CAAGCCTGCACCACCACCAC-3′	1.99
*CsEIN3*	XM_004144061.2	5′-GCACCTGCTCGGCTGATGAAC-3′	5′-GGCAGTTGTTGCTGTTGGTTGTG-3′	1.96
